# First Full Genome Sequence of a Human Enterovirus A120, Isolated in Madagascar

**DOI:** 10.1128/genomeA.00568-14

**Published:** 2014-06-19

**Authors:** Richter Razafindratsimandresy, Marie-Line Joffret, Francis Delpeyroux, Jean-Michel Heraud

**Affiliations:** a Unité de Virologie, Institut Pasteur de Madagascar, Antananarivo, Madagascar; b Institut Pasteur, Paris, France; c INSERM U994, Institut National de la Santé et de la Recherche Médicale, Paris, France

## Abstract

We report the first complete genome sequence of an enterovirus isolate belonging to the human enterovirus A species of the *Picornaviridae* family and to type A120 (EV-A120). The EV-A120 isolate MAD-2741-11 was obtained from the stool of a healthy child living on Madagascar Island. The isolate genome was amplified by a reverse transcription-PCR method, and the consensus sequence was determined.

## GENOME ANNOUNCEMENT

The genus *Enterovirus* in the family *Picornaviridae* comprises related viruses associated with either asymptomatic infection or various clinical syndromes, including respiratory illness, gastroenteritis, meningitis, encephalitis, and paralytic syndromes (1). Seven enterovirus (EV) species are known to infect humans: human enteroviruses (HEV) A, B, C, and D and human rhinoviruses A, B, and C (see http://www.picornaviridae.com/enterovirus/enterovirus.htm). They are classified according to their nucleotidic identity and peptidic similarity rates within the VP1 region (2, 3). Enteroviruses are small, nonenveloped viruses. The RNA genome is an ~7.5-kb single-stranded, positive-sense, polyadenylated molecule, with a single long open reading frame (ORF) flanked by 5′ and 3′ untranslated regions (UTRs) (4). This report describes the sequence of an EV-A120 isolate belonging to a new enterovirus type characterized from a partial genome sequence and recently reported (GenBank accession no. KF700245 [also see http://www.picornaviridae.com/enterovirus/ev-a/ev-a.htm]).

In Madagascar, isolate MAD-2741-11 was obtained from the stool sample of a 3-year-old healthy child living in Toliara, a district located on the southwestern part of the island. This isolate induced a cytopathic effect in both human rhabdomyosarcoma (RD) and human larynx epidermoid carcinoma (HEp-2c) cell lines. Partial overlapping sequences of all genomic regions were obtained using degenerate primers as previously described and a genome-walking method (5–8). The full genome sequence of isolate MAD-2741-11 was reconstructed and aligned with those of other HEV-A strains using ClustalW. Phylogenetic trees corresponding to different genomic regions were constructed using MEGA 5.2 (9).

The genome organization of isolate MAD-2741-11 is similar to those of the previously reported EV genomes. The full genome is 7,407 bp in length, excluding the polyadenylated tract. The G+C percentage for the whole genome is 47.5%. The 5′ UTR is 748 nucleotides (nt) long, and the 3′ UTR is 83 nt long. A large open reading frame (6,576 nt) encodes a polyprotein precursor of 2,191 amino acids.

The whole VP1 region (885 nt) of isolate MAD-2741-11 was compared with those of other enterovirus isolates and prototype strains available in nucleotidic sequence databanks. Blast comparison and phylogenetic analysis showed that the VP1 sequence of isolate MAD-2741-11 was closely related to the first and only reported VP1 sequence of the EV-A120 isolate 46402 from Tajikistan (GenBank accession no. KF700245). The two VP1 sequences shared 81.0% nt and 94.6% amino acid identities, in agreement with values for isolates belonging to the same enterovirus type (8). The P2 and P3 genomic sequences of isolate MAD-2741-11 shared, respectively, 82% and 88% nt identities with those of one of the closest enteroviruses of species A, the coxsackievirus A10 isolate CVA10/SD/CHN/09 (GenBank accession no. HQ728262). The whole polyproteins of the two isolates shared 86% amino acid identity, in agreement with values for isolates belonging to identical enterovirus species (3). These data indicated that isolate MAD-2741-11 can be unambiguously characterized as an EV-A120 isolate. To our knowledge, this work describes the first full genome sequence of this type of human enterovirus.

### Nucleotide sequence accession number.

The genome sequence of the isolate MAD-2741-11 has been deposited in the ENA database as that of an EV-A120 isolate under the accession no. LK021688.
